# Physiology of the Salivary Glands

**Published:** 1880-08

**Authors:** S. M. Prothro

**Affiliations:** Chattanooga, Tenn


### THE DENTAL REGISTER.

VOL. XXXIV.] AUGUST, 1880. [No. 8.

### THE PHYSIOLOGY OF THE SALIVARY GLANDS AND THEIR SECRETIONS.

*Read before the Tennessee Dental Association.*

BY S. M. PROTHRO, OF CHATTANOOGA, TENN.

MR. PRESIDENT AND GENTLEMEN OF THE ASSOCIATION:—When, about three weeks since, I was informed by the Ex. Committee of this Association, that I had been selected to prepare and read a paper before this body, "On the Physiology of the Salivary Glands and their Secretions," my first impression was, that, though the thought of some dainty tempting dish may cause a free flow of saliva, there was nothing in this subject calculated to inspire a free or interesting flow of thought. But when I sat down and began to meditate upon, and associate these glands with the mouth, and observe the office they perform in this cavity alone, I soon realized the fact that I had been assigned a very important and interesting subject.

Let us lop off for a while from the human organism these salivary glands, and see what would be the result, in a short space of time the tongue would cleave to the roof of the mouth, and this so-called, unruly member, ever before so prolific in good or bad words, without the lubricating effect of these secretions would be powerless to act. We could not by words make known our simplest wants, the fondest emotions of the soul would fail to find utterance, the lover incapable of whispering even in the ear of his idol, the devotion of his heart, nor she reply in accents soft as the breath of the vernal morn to his wooing, no sound of sweet music to swell our breast with joy and gladness, for the tongue is stiff and still, the mouth too, dry and parched, would render the process of mastication not one of pleasure and extreme gratification as at present, but tedious and painful, we would not hail with delight the sound of the bell calling us to our meals, but, would shrink from partaking them as we would the most nauseous medicines, for copious drafts of water even would signally fail to moisten and prepare our food for being swallowed, much less for being digested. The suppression of the salivary secretions would entail as deleterious effect upon the general system as the stoppage of the billiary, urinary or any other secretions.

From Zell's Encyclopedia we will give the definition of saliva: "The saliva from sal. salt, that fluid by which the mouth and tongue are constantly moistened in their natural state, and which is supplied by glands which form it, called the salivary glands—the parotid, submaxillary and sublingual. The saliva itself has neither color nor smell, and is tasteless; for although it contains a little salt, the nerves of the tongue are so accustomed to it that it is imperceptible. Its specific gravity is somewhat greater than that of water, and it is supposed that about 12 pounds are secreted in 12 hours. From the mechanical pressure of the muscles upon the salivary glands, the secretion is much augmented during speaking and mastication. In hungry persons too, it is largely secreted at the sight of agreeable food, its uses are to augment the taste of food by the evolution of sapid matter to mix with, dissolve and resolve into its principles the food during mastication, so as to change it into a pultaceous mass fit to be swallowed, to moderate thirst, by moistening the cavity of the mouth and fauces. In its healthy state it consists of at least four-fifths of water, comprising besides mucilage, albumen, muriates of soda, phosphate of soda, phosphate of lime and phosphate of ammonia."

The mouth is the irregular cavity which contains the organs of taste, and the principal instruments of mastication. There are six glands communicating through their excretory ducts with the mouth, as before stated.

The parotid gland from two Greek wórds meaning near the ear, the largest of the three, "is situated directly in front of the ear, and extends over the masseter muscle for a short distance, superficially and deeply behind the ramus of the lower jaw, it reaches below the level of the angle of the lower jaw, and posteriorly to the mastoid process. Embedded in its substance are the external carotid artery, temporo-maxillary vein and facial nerve, the transverse facial artery and branches of the pes anserinus emerge from its anterior border and above the temporal artery, its main duct, called Stenon's duct from Nicholas Stenon, an anatomist who discovered it commences at the papilla upon the internal surface of the cheek, opposite the second molar tooth of the upper jaw and piercing the buccinator muscle, crosses the masseter to the anterior border of the gland where it divides into several branches, which subdivide and ramify through its structure, the area of the canal of this duct is quite small, though it is remarkably thick and dense."

"The submaxillary gland is situated in the posterior angle of the submaxillary triangle of the neck, it rests upon the mylo-hyoidens and hyo-glossus muscles and is covered in by the body of the lower jaw and by the deep cervical fascia, it is separated from the parotid gland by the stylo-maxillary ligament and from the sublingual by the mylo-hyoidens muscle, the facial artery and the submaxillary ganglion are embedded in its lobular substance. Wharton's excretory duct of this gland, commences at the side of the fræuum linguæ and passes backward beneath the mylo-hyoidens, and resting upon the hyo-glossus muscle to the middle of the gland where it ramifies with the structure by numerous branches."

"The sublingual is an elongated and flattened gland, lying beneath the mucous membrane of the floor of the mouth on each side of the frænum linguæ, it pours its secretion into the mouth by seven or eight small ducts, which open by small apertures on each side of the frænum linguæ."

"The salivary glands are conglomerate, consisting of lobes which are made up of polyhedral lobules, and these of smaller lobules. These glands are also abundantly supplied with blood vessels and nerves." The parotid by the external carotid artery, and by nerves derived from the auricular branch of the inferior maxillary, from the auricularis magnus and from the nerve molles, accompanying the external carotid artery.

"The submaxillary gland is supplied by the facial artery and by branches of the submaxillary ganglion, and filaments of the mylo-hyoidian nerve."

"The sublingual gland is supplied by the sublingual branch of the lingual artery, and by filaments of the submaxillary ganglion and gustatory nerve."

The first of the series of changes to which the food is subjected in the digestion canal, takes place in the cavity of the mouth, the solid articles of food are here submitted to the action of the teeth, where they are crushed and divided, and by being mixed with the fluids of the mouth are reduced to a soft pulp capable of being easily swallowed. The fluids with which the food is mixed in the mouth, consist not only of the secretion of the salivary glands, but the mucus secreted by the lining membrane of the whole buccal cavity.

"The glands concerned in the production of saliva are very extensive, and in man and mammalia generally are presented in the form of the three pairs already enumerated and the intra linguæ and numerous smaller bodies of similar structure and with separate ducts, which are scattered thickly beneath the mucous membrane of the mouth, lips, cheeks, soft palate and root of the tongue."

Saliva as it commonly flows from the mouth is mixed with the secretion of the mucous membrane and often with air bubbles, which by its viscidity and being retained make it frothy. "When obtained from the parotid ducts and free from mucus, saliva is a transparent watery fluid with a specific gravity of 1006 to 1009, and in which when examined by the microscope are found floating a number of minute particles, derived from the secreting ducts and vessicles of the glands." In impure or mixed saliva are found besides these particles, numerous epithelial scales, separated from the mucous membrane of the mouth and tongue, and mucous corpuscles discharged mostly from the tonsils.

Physiology teaches us that in reaction the saliva when first secreted is always alkaline, and that from the parotid gland is said to be more strongly alkaline than that from any other of the salivary glands. This alkaline condition seems to be most evident when digestion is going on, and the degree of alkalinity bears a direct proportion to the acidity of the gastric fluid secreted at the same time. During fasting the saliva though secreted alkaline soon becomes acid, especially if secreted slowly and allowed to mix with the acid mucus of the mouth, by which its alkaline reaction is destroyed.

"The principal organic constituents of normal saliva are albumen, ptyalin and mucin, the mucin gives to saliva its viscidity, while ptyalin is its fermentable principle." The acids which are incorporated in the saliva are hydrochloric, nitric, sulphuric, carbonic, acetic, oxalic, uric and lactic." The alkalinity of the saliva is doubtless due to the presence of the salts of lime and soda in the form of phosphates. The salivary calculous which is so often found deposited upon the teeth, is composed of phosphate of lime with other organic matter. There are other substances that may be detected in the saliva, such as cholestrine, sulpho-cyanide of potash, (which is a secretion of the sublingual gland,) besides grape sugar, and even pus cells and blood corpuscles are discovered by the microscope.

It is truly wonderful what chemical changes are constantly going on in this laboratory, the oral cavity. The most powerful acids, such as nitric and sulphuric are elaborated from the elements contained in the mouth and saliva, which exert such an active part in the destruction of our teeth, sulphuretted hydrogen which we may at times detect in the breath of our patient, is dissolved by the saliva, when its elements go through a process of oxidation and the consequent formation of sulphuric acid, the active cause of black decay. White decay is caused by the formation of nitric acid from the ammonia in the fluids of the mouth. The elements of ammonia, nitrogen and hydrogen, are oxidized when exposed to the oxygen contained in the bubbles of air that float in the saliva. This oxidation forms nitric acid in its nascent state, which is the active agent of white decay, the ammonia may be detected in the breath of the patient as well as by a ropy condition of the saliva.

We will notice the chemical changes that take place in the mouth in the production of black decay, this decay differs in other respects, as well as in color, from the other forms of dental decay. The tissues are much less disintegrated, the lime salts are not dissolved to that extent as in the other varieties, the lime is still present, but not in the same combination as in the natural tooth substance. It is interesting to observe the striking contrast between the processes that take place in this and the ordinary decay, hydrochloric acid the destroying agent in the other variety acting on the carbonate of lime, forms chloride of calcium, while at the same time it freely dissolves the bone phosphate, but sulphuric acid, the destructive agent in black decay acting on the carbonate gives sulphate of lime, or plaster of paris, a salt almost insoluble; and this acid almost totally fails to either dissolve or decompose the phosphate. The organic matter of the tooth is converted into animal charcoal, which accounts for its black color.

The ashes of saliva have been analyzed by Enderlin, who found that they consist of substances very similar to those in the ashes of blood, and he believes that the alkalinity of the saliva, like that of the blood is due to the tribasic phosphate of soda, the other salts which he found in it were chlorides of sodium and potassium, sulphate of soda and phosphate of lime, magnesia and iron, saliva is also said to contain a small quantity of sulpho-cyanogen, the presence of which is indicated by a deep red color when the saliva is mixed with a neutral solution of a salt of the per-oxide of iron. The rate at which saliva is secreted is subject to considerable variation. When the tongue and muscles concerned in mastication are at rest, and the nerves of the mouth are not sujected to unusual stimulus, the quantity secreted is not more than sufficient, with the mucus to keep the mouth moist, but the flow is much accelerated, when the movements of mastication take place and especially when they are combined with the presence of food in the mouth. Mental impressions too, even when the mouth is at rest, produced by the sight or thought of food may excite it, the quantity of saliva under these varying circumstances secreted in 24 hours varies also, its average amount probably ranges from fifteen to twenty ounces.

Mitscherlich found in a man who had a fistulous opening in the parotid duct that the quantity discharged in twenty-four hours was from two to three ounces; and the saliva collected from the mouth and derived from the other salivary glands amounted to six times more than from the one parotid.

The purposes served by saliva are of several kinds, acting mechanically, in the first place, it keeps the mouth in a moist condition, facilitating the movements of the tongue in speaking and in the mastication of food, it also thus serves in dissolving sapid substances and rendering them capable of exciting the nerves of tastes, but the principal mechanical purpose of the saliva is that by mixing with the food during mastication, it makes it a soft pulpy mass so that it may be easily swallowed, it is adapted to this purpose both as to quality and quantity, generally speaking, the quantity secreted during a meal is in direct proportion to the dryness and hardness of the food.

M. Lassaigne has shown by a table the quantity produced in the mastication of a hundred parts of each of several kinds of food; thirty parts suffice for a hundred parts of crumbs of bread, but not less than 120 for the crust, 42.5 parts of saliva are produced for the hundred of roast meat, 3.7 for as much of apples, and so on, according to the general rule above stated, the quality of saliva is equally adapted to this end, it is easy to see how much more readily it mixes with most kinds of food than water alone does; and M. Bernard believes from his experiments that the saliva from the parotid, labial and other small glands being more aqueous than the rest, is that which is chiefly braided and mixed with the food in mastication, while the more viscid mucoid secretion of the submaxillary, palatine and tonsillitic glands is spread over the surface of the softened mass to enable it to slide more easily through the fauces and æsophagus.

There are reasons for supposing, beyond these, its mechanical purposes, that saliva performs some chemical part in the digestion of the food. The chief of these reasons are, the number and size of the glands engaged in the secretion; the variety of substances which enter into its composition, and which can scarcely be supposed to be of use so far as its mechanical properties are concerned: The quantity which is secreted not only during mastication, but after the food has passed into the stomach, especially in old persons, who from loss of their teeth frequently swallow their food in an imperfectly masticated state; the fact that the saliva secreted during digestion is more alkaline than at other times; and lastly the result of certain experiments.

Among the experiments are those of Spallanzain and Reoumar, who found that food enclosed in perforated tubes and introduced into the stomach of an animal, was found more quickly digested when it had been previously impregnated with saliva, than when it was moistened with water. Dr. Wright also found that if the æsophagus of a dog is tied and food mixed with water alone is placed in the stomach, the food will remain undigested though the stomach may secrete abundant acid fluid; but if the same food were mixed with saliva and the rest of the experiment similarly performed—the food was readily digested. It is, however, uncertain as to the precise nature of the chemical part the saliva takes, though it may appear that it exerts more than a mechanical influence in promoting digestion.

As traced by chemical analysis its composition offers no guide, its alkalinity is never sufficient to neutralize the gastric fluid, though it appears to increase in the same proportion as the acidity of the secretion of the stomach, both in health and disease, the contents of the stomach, including as they do the saliva swallowed are always acid, the saliva remains in contact with the food so short a time before it is neutralized by the acid of the stomach, that the idea is precluded that the alkali is the principal constituent by which it assists in digestion, however, its organic principles may have more power, as shown by experiment, that when saliva, or a portion of salivary gland is added to starch paste, the starch is promptly transformed into dextrine and grape sugar, and when common raw starch is masticated and mingled with saliva, and kept with it at a temperature of 90 or 100°, the starch grains are cracked or eroded and their contents are transformed in the same manner as the starch paste. Changes similar to these are effected on the starch of farinaceous food, (especially after cooking) in the stomach, and it is reasonable to refer them to the action of the saliva, because the acid of the gastric fluid tends to retard or prevent rather than favor the transformation of the starch. We therefore hold that a purpose served by the saliva in the digestive process is that of assisting in the transformation of the starch, which enters so largely into the composition of most articles of vegetable food, and which being naturally insoluble, is converted into the soluble dextrine or grape sugar, and made fit for absorption.

Magendie has demonstrated by experiment that many substances besides saliva may excite the conversion of starch into grape sugar, such as pieces of mucous membrane of the mouth, bladder, rectum and other parts, but the gastric fluid will not produce the same effect. The property therefore cannot be assigned to any peculiar organic principle in the saliva, the part of the saliva which appears most active is that secreted by the small glands, and the mucous membrane of the mouth.

We will now stop to enquire if the influence of the saliva in assisting the digestion of farinaceous food be admitted, and that point does not seem to be disputed, is there a corresponding purpose served by the saliva of the carnivora who use no such food? There is but little information at present gathered upon this point, it has not been ascertained whether or not saliva exercises any kind of digestive power over animal substances. I would feel delighted to be able to give the desired information in this paper, but as our literature is silent and I have not been prepared to make any test, we will have to let the matter stand in abeyance until some of our solons favor us with more light upon the subject. We will give some of the tests by which we may detect the various acids and substances found in the saliva, and we are done.

The chemical test for hydrochloric acid, is, add a little saliva in a test tube to a few drops of a solution of nitrate of silver, if this acid be present a white precipitate will be thrown down, insoluble in acid and soluble in ammonia.

Test for sulphuric acid.—Add a solution of chloride of barium, a white precipitate, insoluble in acids and alkalies.

Test for nitric acid.—Add to a small portion of sulphuric acid in a test tube, some ferrous sulphate, then drop in some saliva and a brown ring will form.

Test for phosphate.—Add a solution of uric acetate and sodic acetate, a white precipitate.

Test for uric acid.—Evaporate a small portion of ammonic hydrate, you will get a crimson color.

Test for urea.—Add to a small portion of saliva on a glass slide nitric acid, crystals of nitrate of urea may be seen with the microscope, they are rhombic plates, soluble in water.

Test for urates.—Decompose by nitric acid, and apply test for uric acid.

Test for carbonic acid.—Add acetic acid and examine under the microscope for effervescence of carbonic anhydride.

Test for oxalic acid.—Add solution of calcium and oxalate of calcium, octahedral crystals will be detected under the microscope.

Test for ferro-cyanide of potash.—Add ferri chloride and a blood red color will result, which will be destroyed by adding in solution corrosive sublimate.

Test for albumen.—Heat and it will coagulate, this is insoluble in acids.

Test for ptyaline.—Digest parotid gland of ox or sheep 24 hours, strain and put in glycerine and strain again, ptyaline will appear in white flakes.

Test for cholestrine.—When pure, if sulphuric acid be added, you will get a play of colors, it is soft like albumen, soluble in hot alcohol, deposited when cold in flat flakes.

In conclusion we suggest that it may be pleasurable as well as profitable to us to keep on hand the chemicals and appliances, (for they cost but little,) and as occasion may offer, examine the condition of the saliva of our patients, we trust too much to the thinking and statements of others and are apt to fall into error in accepting untried theories as truths, old theories are being uprooted year after year, and new and entirely adverse ones succeed them. This is an age of investigation, and when I

reflect upon what has been accomplished in our profession in the last decade by a few patient and careful laborers, who have devoted their valuable time to speed dentistry onward to its legitimate position as a specialty of general medicine, I cannot but feel a self reproach at the small part I have taken toward the attainment, in not a remote future of the acme of our wishes and hopes. There is some specific work for every one of us to perform. He who lives the life of a drone and wastes his talents and gives nothing to the human family, either in point of time, attention, teaching, discovery or observation, has in the language of another, "deliberately cast aside all that is divine, and placed himself on the platform with the brutes." On the other hand, he that is giving his time, attention and the results of his application and observation is doing all that he can to benefit humanity, and consequently deserves his reward; if he has discovered nothing brilliant or startling in his life of usefulness to give and transmit to posterity, it has not been his fault, and often these bright and startling discoveries are the result of mere fortuity, still when his career is run and his almond tree has flourished, his grinders have ceased because they are few, and the windows are darkened and having to lay down the mallet and the gold, he will experience the conciousness of having lived for a purpose other than the aggrandizement of self, and his patrons and the community in which he lived will accord him the epithet, "will done thou good and faithful servant, go up higher and receive thy reward."

